# Supplementary material for: Acoustofluidic-based therapeutic apheresis system
Source: Nat Commun. 2024 Aug 10;15:6854. doi: 10.1038/s41467-024-50053-1 (PMC11316742; doi:10.1038/s41467-024-50053-1)
Supplement: Supplementary file 1 — Supplementary Information [file 41467_2024_50053_MOESM1_ESM.pdf]

Supplementary Materials

Acoustofluidic-based Therapeutic Apheresis System

Mengxi Wu[1, 2]†, Zhiteng Ma[2]†, Xianchen Xu[2], Brandon Lu[3], Yuyang Gu[2], Janghoon Yoon[4], Jianping Xia[2], Zhehan Ma[3], Neil Upreti[3], Imran J. Anwar[4], Stuart J. Knechtle[4], Eileen T. Chambers[4], Jean Kwun[4,*], Luke P. Lee[5,6,7,8,9,*], Tony Jun Huang[2,*]

[1]School of Mechanical Engineering, Dalian University of Technology, Dalian, Liaoning, P.R. China

[2]Thomas Lord Department of Mechanical Engineering and Materials Science, Duke University, Durham, NC 27708, USA

[3]Department of Biomedical Engineering, Duke University, Durham, NC 27708, USA

[4]Duke Transplant Center, Department of Surgery, Duke University Medical Center, Durham, NC 27708, USA

[5]Harvard Medical School, Harvard University; Renal Division and Division of Engineering in Medicine, Department of Medicine, Brigham and Women's Hospital, Boston, MA 02115, USA

[6]Department of Bioengineering, University of California, Berkeley, Berkeley, CA 94720, USA

[7]Department of Electrical Engineering and Computer Science, University of California, Berkeley, Berkeley, CA 94720, USA

[8]Department of Biophysics, Institute of Quantum Biophysics, Sungkyunkwan University, Suwon, Korea

[9]Department of Chemistry and Nanoscience, Ewha Womans University, Seoul, Korea

†These authors contributed equally to this work.

Correspondence and requests for materials should be addressed to J. K., L.P.L and T.J.H. (email: jean.kwun@duke.edu; lplee@bwh.harvard.edu; tony.huang@duke.edu)

Note S1: Simulation models

We have incorporated two sets of fluid stabilizers into the circulation system to effectively tackle challenges stemming from fluctuating blood pressure and the instability induced by peristaltic pumps. Each fluidic stabilizer consists of four cavities connected to the main fluid microchannel. Supplementary Fig. 1 illustrates the cross-section view and corresponding models of these fluids.

As shown in Supplementary Fig. 1, when in steady state, the pressure in the nth conjunction of side-channel 2 and the main channel is p_n, with a flow rate of $Q_{a,n}$ in the main channel. Due to Poiseuille's Law, and according to the Euler equation (*1*)

$$\frac{dQ_{a,n}}{dt}\rho l_{1,n} = \left(p_n - Q_{a,n}R_{1,n}\right)A_{1,n} \tag{1}$$

where ρ is the density of the fluid, $A_{1,n}$ is the cross-section area of the nth main channel, $l_{1,n}$ is the length of the nth stabilizers on the main channel, and $R_{1,n}$ is the flow resistance of the nth main channel. During the stabilization process in the channel, we assume that the flow rate of the outlet increases by $\Delta Q_{out,n}$ when the pressure changes by Δp_n. Thus, the force balance near the outlet of the channel can be expressed as

$$\frac{d(Q_{a,n}+\Delta Q_{out,n})}{dt}\rho l_{1,n} = \left(p_n + \Delta p_n - ((Q_{a,n} + \Delta Q_{out,n})R_{1,n}\right)A_{1,n} \tag{2}$$

After substituting Eq. (1) into the Eq. (2), the force balance near the outlet can be rewritten as

$$\frac{d(\Delta Q_{out,n})}{dt}\rho l_{1,n} = \left(\Delta p_n - (\Delta Q_{out,n})R_{1,n}\right)A_{1,n} \tag{3}$$

The side channel can thus be considered as a mass-spring system. The total mass inside of the cavity can be assumed to be a constant. Considering that the volume change is much smaller than the cavity volume and that the temperature reaches steady state in less than 200 μs in ambient area (γ = 1), The pressure inside of the nth side channel can be expressed as (*2*)

$$p_n = p_{2,n}\left(V_{2,n}/(V_{2,n} - \Delta V_n)\right)^{\gamma} \tag{4}$$

Which can also be rewritten as

$$p_n = p_{2,n}\left(1/(1 - \frac{\Delta V_n}{V_{2,n}})\right) \tag{5}$$

Where $\Delta V_n = A_{2,n}\Delta l_{2,n}$ represents the volume change inside of the side channel, and $V_{2,n}$and $p_{2,n}$ represent the initial pressure and volume of the trapped air in the side channel and cavity, respectively.

Since $-1 < \frac{\Delta V_n}{V_{2,n}} < 1$, The Eq. (6) can be rewritten as Taylor series form.

$$p_n = p_{2,n} \sum_{N=0}^{\infty} (-1)^N (-\Delta V_n / V_{2,n})^N \,, N \in \mathbb{Z}, with \; -1 < \frac{\Delta V_n}{V_{2,n}} < 1 \qquad (6)$$

Which can be expressed as

$$p_n = p_{2,n}(1 + \frac{\Delta V_n}{V_{2,n}} + \cdots (-1)^N \left(-\frac{\Delta V_n}{V_{2,n}}\right)^N) \,, \; N \in \mathbb{Z}, with \; -1 < \frac{\Delta V_n}{V_{2,n}} < 1 \qquad (7)$$

Since the change in volume is much smaller than cavity volume ($\Delta V_n \ll V_{2,n}$), we neglect the higher order terms of the Taylor series, yielding

$$p_n = p_{2,n}(1 + \frac{\Delta V_n}{V_{2,n}}) \qquad (8)$$

Thus, by considering the forces of the flow column, the force balance inside the side channel connected to the cavity can be expressed as

$$\frac{d(\Delta Q_{2,n})}{dt} \rho l_{2,n} = \left(\Delta p_n - \Delta Q_{2,n} R_{2,n}\right) A_{2,n} + (p_n - p_{2,n}) A_{2,n} + \rho g A_{2,n} \Delta l_{2,n} \qquad (9)$$

where $p_{2,n}$ is the initial pressure, $V_{2,n}$ is the initial volume of the trapped air, ρ is the density of fluid, $A_{2,n}$ is the cross-section are, $\Delta l_{2,n}$ is the flow column increasement, and $R_{2,n}$ is the flow resistance of the nth side channel, respectively. $Q_{2,n}$ is the flow rate in the side channel. $\Delta Q_{2,n}$ is the increase in flow rate in the side channel, which has the following connection with the inlet and outlet flow rate:

$$\Delta Q_{in,n} = \Delta Q_{out,n} + \Delta Q_{2,n} \qquad (10)$$

Where the inlet and outlet flow rate have the following equations by considering the total flow rate in the main channel

$$Q_{in,n} = Q_{a,n} + \Delta Q_{in} \qquad (11)$$

$$Q_{out,n} = Q_{a,n} + \Delta Q_{out} \qquad (12)$$

By applying the Laplace transformation to Eqs. (3) and (9), the pressure change inside of the nth main channel and side channel can be expressed as

$$\Delta p_n = \frac{\rho l_{1,n} \Delta Q'_{out,n}}{A_{1,n}} s + \Delta Q'_{out,n} R_{1,n} \qquad (13)$$

$$\Delta p_n = \frac{\rho l_{2,n} \Delta Q'_{2,n}}{A_{2,n}} s + \Delta Q'_{2,n} R_{2,n} - \frac{\Delta V'_n}{V_{2,n}} p_{2,n} A_{2,n} - \rho g A_{2,n} \Delta l_{2,n} \qquad (14)$$

Since $\Delta V_n = \Delta l_{2,n} A_{2,n} = \int \Delta Q_{2,n}/A_{2,n} dt$, Eq. (14) can be expressed as

$$\Delta p_n = \frac{\rho l_{2,n} \Delta Q'_{2,n}}{A_{2,n}} s + \Delta Q'_{2,n} R_{2,n} - \frac{\Delta Q'_{2,n}}{V_{2,n} s} p_{2,n} - \rho g A_{2,n} \Delta l_{2,n} \tag{15}$$

Where $\Delta Q'_{out,n}$, $\Delta Q'_{2,n}$ represent the Laplace term.

Hence, the relationship between the volume flow rate out of the nth main channel and the volume flow rate inside the nth side channel can be expressed by the following equations:

$$\frac{\rho l_{1,n} \Delta Q'_{out,n}}{A_{1,n}} s + \Delta Q'_{out,n} R_{1,n} = \frac{\rho l_{2,n} \Delta Q'_{2,n}}{A_{2,n}} s + \Delta Q'_{2,n} R_{2,n} - \frac{\Delta Q'_{2,n}}{V_{2,n} s} p_{2,n} - \rho g \frac{\Delta Q'_{2,n}}{s A_{2,n}} \tag{16}$$

and

$$\Delta Q'_{out,n} = \epsilon \Delta Q'_{2,n} \tag{17}$$

where

$$\epsilon = \frac{1}{\eta_0} \left(\eta_1 - \frac{p_{2,n}}{V_{2,n} s}\right) \tag{18}$$

and where

$$\eta_0 = \frac{\rho l_{1,n}}{A_{1,n}} s + R_{1,n} \tag{19}$$

$$\eta_1 = \frac{\rho l_{2,n}}{A_{2,n}} s + R_{2,n} - \rho g \frac{1}{s A_{2,n}} \tag{20}$$

As shown in Supplementary Fig. 1, the fluid stabilizers are periodically attached to the main channel. We can assume that the initial parameters for each stabilizer are the same. Thus, the increase in flow rate in the nth outlet can be expressed as

$$\Delta Q'_{out,n} = \frac{1}{\eta_0} \left(\eta_1 - \frac{p_{2,n}}{V_{2,n} s}\right) \Delta Q'_{2,n} \tag{21}$$

Combining Eq. (11) and (12), yields

$$\Delta Q_{in,n} = \Delta Q_{out,n} + \Delta Q_{2,n} \tag{22}$$

By considering the flow rate continuity conditions between the nth main stabilizer and $(n-1)^{th}$ stabilizer, $\Delta Q_{in,n} = \Delta Q_{out,n-1}$, we can deduce that the relationship between the flow rate in the 1st stabilizer and the flow rate in the last stabilizer has the following relation:

$$\Delta Q_{in,1} = \Delta Q_{out,N} + \Delta Q_{2,n} + \Delta Q_{2,n-1} + \cdots + \Delta Q_{2,1} \quad (n = 1,2,3 \ldots, N, N \in \mathbb{Z}) \tag{23}$$

The Laplace transform thus also has the following relationship

$$\Delta Q'_{in,1} = \Delta Q'_{out,N} + \Delta Q'_{2,n} + \Delta Q'_{2,n-1} + \cdots + \Delta Q'_{2,1} \quad (n = 1,2,3 \ldots, N, N \in \mathbb{Z}) \tag{24}$$

Therefore, Eq. (23) and Eq. (24) can be rewritten as

$$\Delta Q_{out,N} = \Delta Q_{in,1} - \sum_{n=1}^{N} \Delta Q_{2,n} \tag{25}$$

And

$$\Delta Q'_{out,N} = \Delta Q'_{in,1} - \sum_{n=1}^{N} \Delta Q'_{2,n} \tag{26}$$

By considering that the inlet flow rate $Q_{in,1}$ has an initial disturbance of $\Delta Q_{in,1}$ from the steady inlet flow rate $Q_{a,1}$, we yield the following equation expression.

$$Q_{in,1} = Q_{a,1} + \Delta Q_{in,1} = Q_{a,1} + Q_{a,1}(\alpha \sin(\omega t)) \tag{27}$$

Where α is the disturbance ratio, and ω is the disturbance frequency.

As shown in Supplementary Fig. 2, the flow rate increase, $\Delta Q_{in,1}$, thus has the following expression.

$$\Delta Q_{in,1} = Q_{a,1}(\alpha \sin(\omega t)) \tag{28}$$

By employing the Laplace transform, the disturbance flow rate can be expressed as

$$\Delta Q'_{in,1} = \int_0^{\infty} \Delta Q_{in,1} e^{-st}\, dt \tag{29}$$

Substituting Eq. (29) into the Eq. (22), then Eq. (22) can be rewritten as

$$\Delta Q'_{out,N} = \int_0^{\infty} \Delta Q_{in,1} e^{-st}\, dt - \sum_{n=1}^{N} \Delta Q'_{2,n} \tag{30}$$

Substituting Eqs. (19) and (20) into the Eq. (30) yields

$$\Delta Q'_{out,N} = \left(\frac{1}{\frac{1}{\eta_0}(\eta_1 - \frac{p_{2,n}}{V_{2,n} s})}\right)^N \Delta Q'_{in,1} \tag{31}$$

And

$$Q\,'_{out,N} = \left(\frac{1}{\frac{1}{\eta_0}(\eta_1 - \frac{p_{2,n}}{V_{2,n} s})}\right)^N \int_0^{\infty} \Delta Q_{in,1} e^{-st}\, dt \tag{32}$$

Thus, the outlet flow rate change can be obtained and expressed as

$$\Delta Q_{out,N} = \left(\frac{1}{\frac{1}{\eta_0}(\eta_1 - \frac{p_{2,n}}{V_{2,n}s})}\right)^N \Delta Q_{in,1} \tag{33}$$

Which can ultimately be rewritten as

$$\Delta Q_{out,N} = \left(\frac{\eta_0}{(\eta_1 - \frac{p_{2,n}}{V_{2,n}s})}\right)^N Q_{a,1}(\alpha\sin(\omega t)) \tag{34}$$

Where,

$$\eta_0 = \frac{\rho l_{1,n}}{A_{1,n}} s + R_{1,n},$$

$$\eta_1 = \frac{\rho l_{2,n}}{A_{2,n}} s + R_{2,n} - \rho g \frac{1}{s A_{2,n}},$$

$$R_{1,n} = \frac{8\pi\mu l_{1,n}}{{A_{1,n}}^2},$$

$$R_{2,n} = \frac{8\pi\mu l_{2,n}}{{A_{2,n}}^2},$$

In Supplementary Fig. 2, the parameters are set as follows: $\rho = 1000kgm^{-3}$, $l_{1,n} = l_{2,n} = 5e^{-3}m$, $A_{1,n} = 0.02e^{-3} * 3e^{-3}m^2, A_{2,n} = \pi R_2^2 = \pi * (0.1e^{-3})^2 m^2$, $V_0 = V_{2,1} = \pi R_3^2 h = \pi * (2e^{-3})^2 * 5e^{-3}m^3$, $p_{2,n} = 1.01e^5 Pa$, $\mu = 8.9e^{-4}$, $s = 10$, $\omega = 10\ rad/s$,

In Fig. S3, the inlet flow rate $Q_{in,1}$, with an initial disturbance of $\Delta Q_{in,1}$ from the steady inlet flow rate $Q_{a,1}$, has a following equation expression.

$$Q_{in,1} = Q_{a,1} + \Delta Q_{in,1} = Q_{a,1} + Q_{a,1}(\alpha_1 \sin(\omega_1 t) + \alpha_2 \sin(\omega_2 t)) \tag{35}$$

with the parameters set as follows: $\rho = 1000kgm^{-3}$, $l_{1,n} = l_{2,n} = 5e^{-3}m$, $A_{1,n} = 0.02e^{-3} * 3e^{-3}m^2, A_{2,n} = \pi R_2^2 = \pi * (0.1e^{-3})^2 m^2$, $V_{2,n} = \pi R_3^2 h = \pi * (2e^{-3})^2 * 5e^{-3}m^3$, $p_{2,n} = 1.01e^5 Pa$, $\mu = 8.9e^{-4}$, $s = 10, \omega_1 = 10\ rad/s$, $\omega_2 = 7\ rad/s$

References

1. Jiao, Z., Zhao, J., Chao, Z., You, Z. and Zhao, J., 2019. An air-chamber-based microfluidic stabilizer for attenuating syringe-pump-induced fluctuations. Microfluidics and Nanofluidics, 23, pp.1-10.
2. Veenstra, T.T., Sharma, N.R., Forster, F.K., Gardeniers, J.G., Elwenspoek, M.C. and van den Berg, A., 2002. The design of an in-plane compliance structure for microfluidical systems. Sensors and Actuators B: Chemical, 81(2-3), pp.377-383.

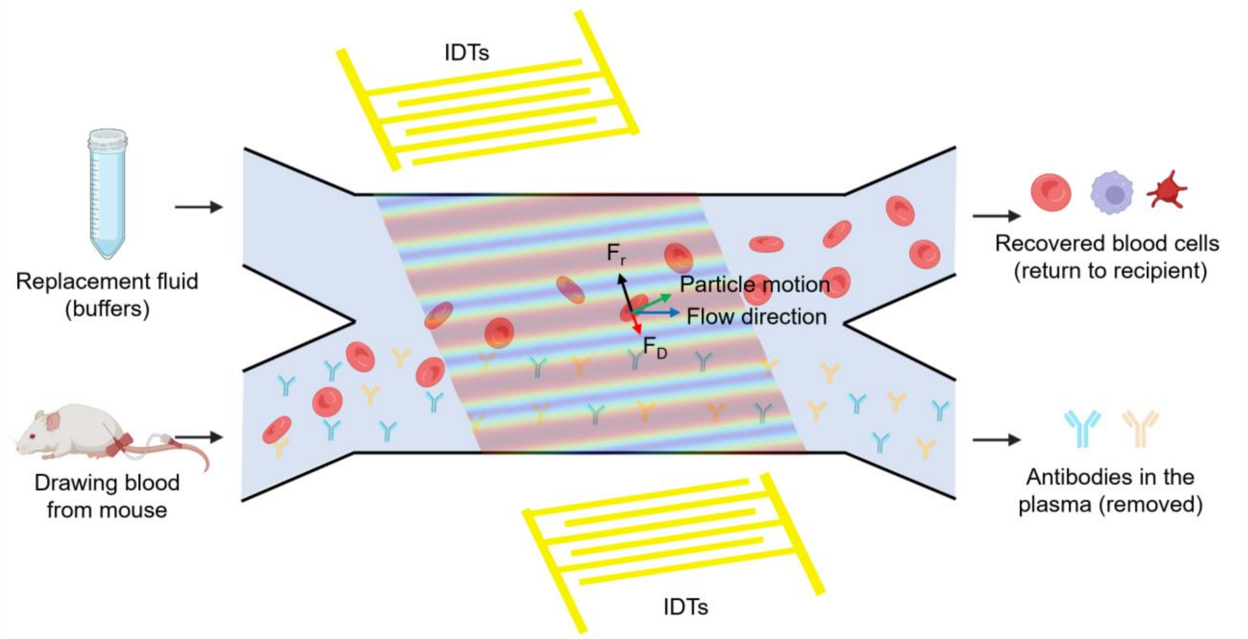

Supplementary Figure 1 | Schematic depicting the mechanism for acoustics-based separation of blood. Figure created with BioRender.com.

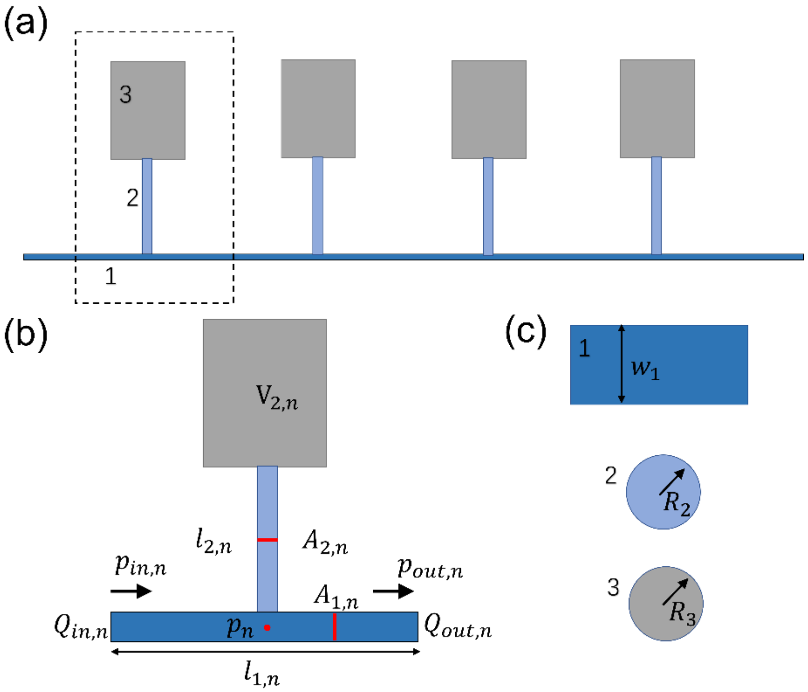

Supplementary Figure 2 | a. Schematic of the flow channel with multiple side chambers. **b.** Unit cell view of nth flow channel with its respective side chamber. **c.** A cross-section view of the unit cell.

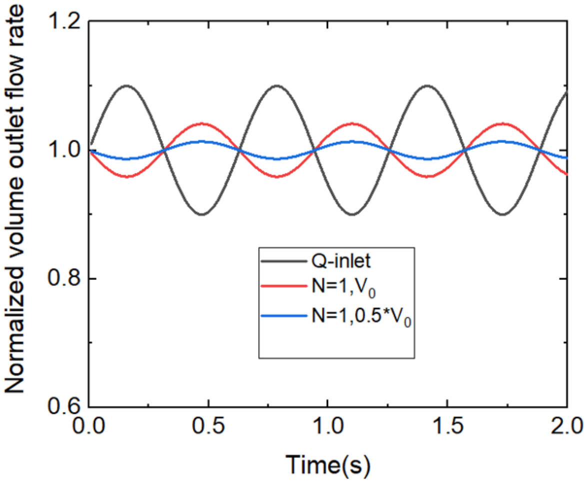

Supplementary Figure 3 | The normalized inlet and outlet volume flow rate with a side chamber volume of V_0 and $0.5\ V_0$.

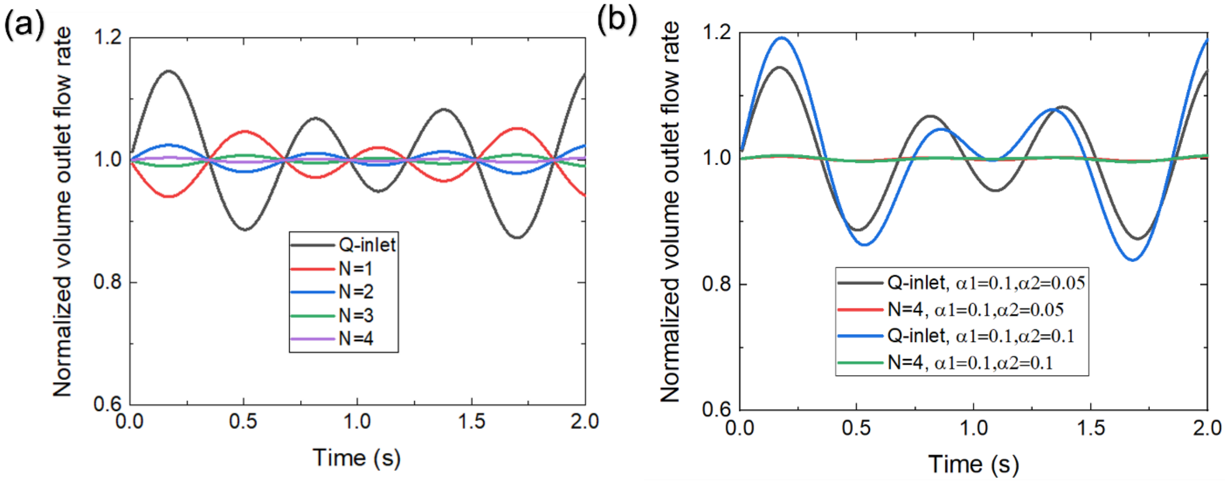

Supplementary Figure 4 | a. The normalized inlet and outlet volume flow rate with a varying number of side chambers (1-4). **b.** The normalized inlet and outlet volume flow rate after passing 4 side chambers. The disturbance ratio $\alpha_1 = 0.1, \alpha_2 = 0.05$ and $\alpha_1 = 0.1, \alpha_2 = 0.1$ respectively.

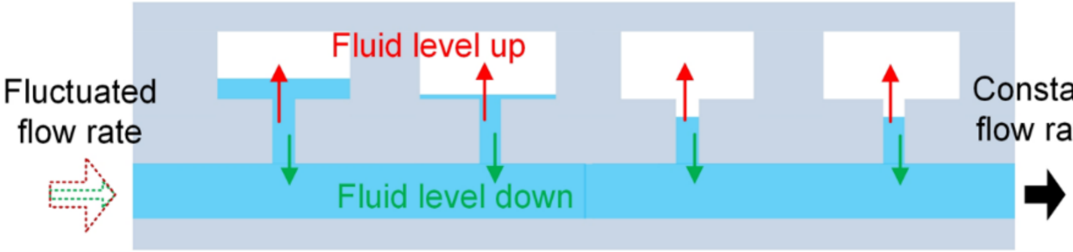

Supplementary Figure 5 | Integration of the four cavities to form a compact series of fluid stabilizers.

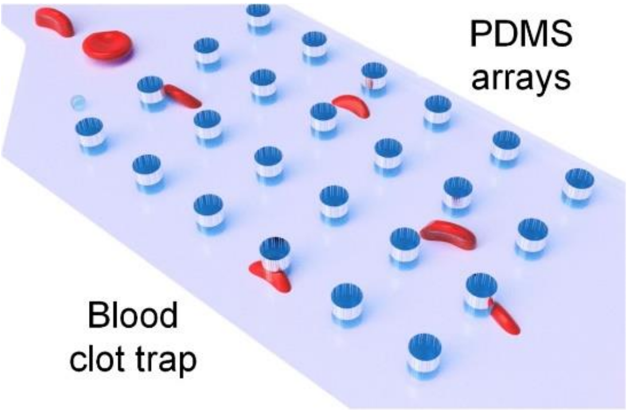

Supplementary Figure 6 | The design of the micropillar array inserted into the microfluidic channel as an on-chip filter.

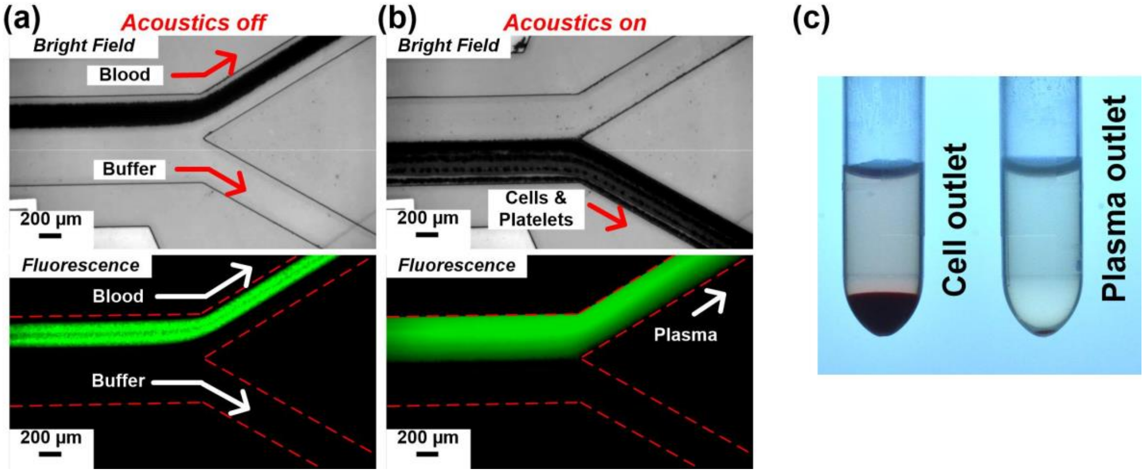

Supplementary Figure 7 | Microscope images of blood flow **a.** when acoustic waves are not activated and **b.** when acoustics are activated. **c.** Samples collected from the cell outlet and plasma outlet when acoustics are on.

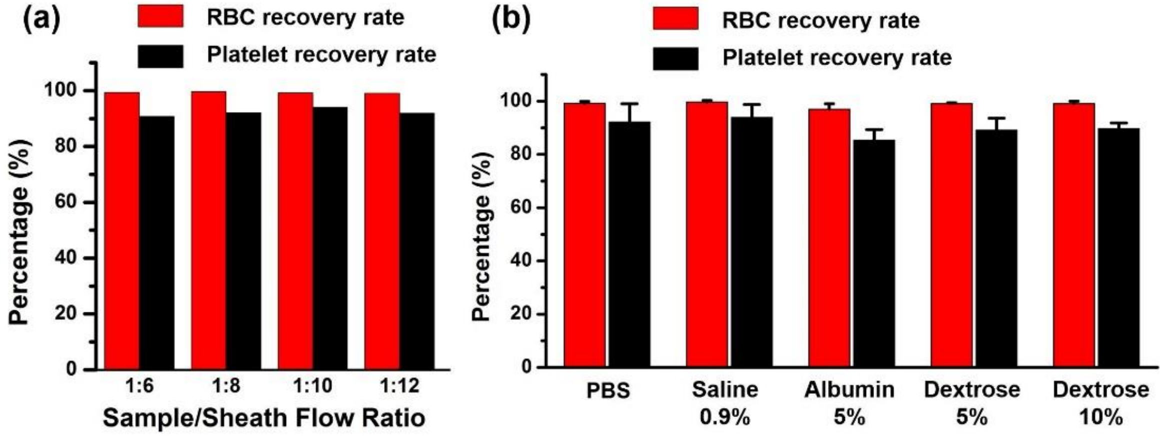

Supplementary Figure 8 | Recovery rates of RBCs and platelets as a function of **a.** sample/sheath flow ratio and **b.** the types of sheath fluid.

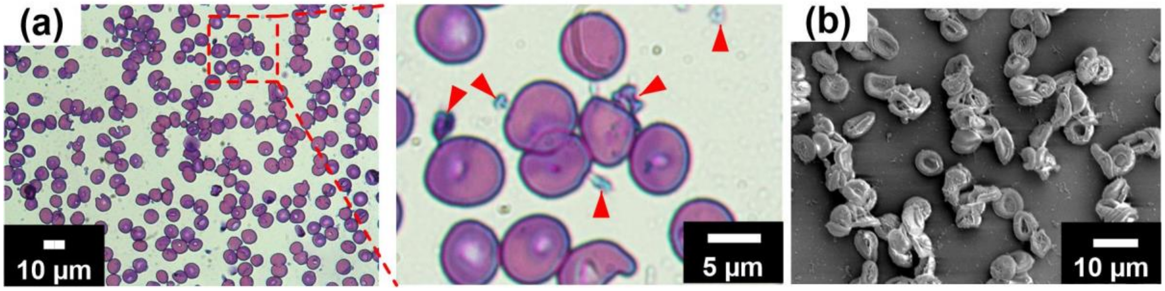

Supplementary Figure 9 | Evaluation of blood components post-separation. a. The Wright staining results showing RBCs and platelets (marked by red arrows) appearing with normal morphology. **b.** SEM images showing the morphology of RBCs.

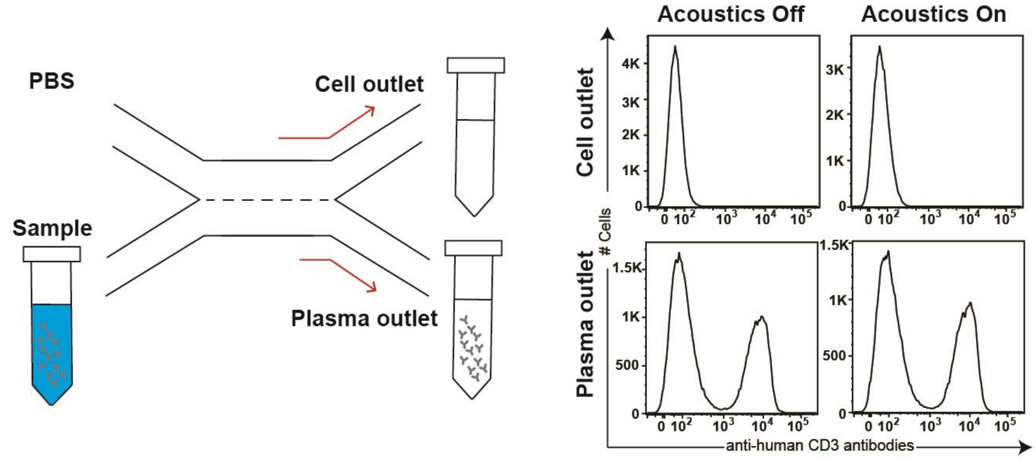

Supplementary Figure 10 | Segregation of immunoglobulin monoclonal antibody (anti-CD3) supplementation with PBS. The unbound antibodies are not altered in the microchip regardless of the presence of acoustic waves and are not diffused to other fractions.

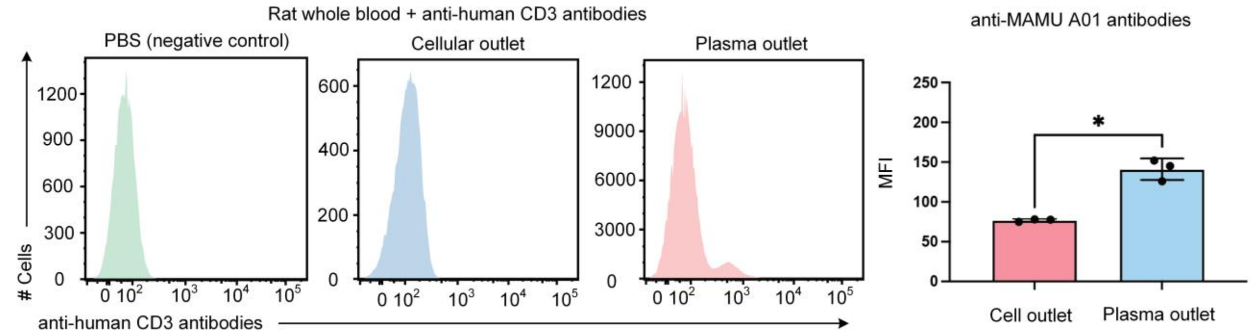

Supplementary Figure 11 | a. Isolation of unbound anti-MAMU A01 antibodies from non-A01 monkey whole blood. **b.** Comparison of the levels of anti-MAMU A01 antibodies present in the plasma outlet and of that in the cell outlet.
