# Supplementary material for: Acoustofluidic-based therapeutic apheresis system
Source: Nat Commun. 2024 Aug 10;15:6854. doi: 10.1038/s41467-024-50053-1 (PMC11316742; doi:10.1038/s41467-024-50053-1)
Supplement: Supplementary file 3 — Reporting summary [file 41467_2024_50053_MOESM3_ESM.pdf]

nature portfolio

Corresponding author(s): Jean Kwun, Luke P. Lee, Tony Jun Huang

Last updated by author(s): Jun 4, 2024

Reporting Summary

Nature Portfolio wishes to improve the reproducibility of the work that we publish. This form provides structure for consistency and transparency in reporting. For further information on Nature Portfolio policies, see our Editorial Policies and the Editorial Policy Checklist.

Statistics

For all statistical analyses, confirm that the following items are present in the figure legend, table legend, main text, or Methods section.

n/a	Confirmed	
☐	☒	The exact sample size (*n*) for each experimental group/condition, given as a discrete number and unit of measurement
☐	☒	A statement on whether measurements were taken from distinct samples or whether the same sample was measured repeatedly
☐	☒	The statistical test(s) used AND whether they are one- or two-sided<br>*Only common tests should be described solely by name; describe more complex techniques in the Methods section.*
☐	☒	A description of all covariates tested
☐	☒	A description of any assumptions or corrections, such as tests of normality and adjustment for multiple comparisons
☐	☒	A full description of the statistical parameters including central tendency (e.g. means) or other basic estimates (e.g. regression coefficient) AND variation (e.g. standard deviation) or associated estimates of uncertainty (e.g. confidence intervals)
☐	☒	For null hypothesis testing, the test statistic (e.g. *F*, *t*, *r*) with confidence intervals, effect sizes, degrees of freedom and *P* value noted<br>*Give P values as exact values whenever suitable.*
☒	☐	For Bayesian analysis, information on the choice of priors and Markov chain Monte Carlo settings
☒	☐	For hierarchical and complex designs, identification of the appropriate level for tests and full reporting of outcomes
☒	☐	Estimates of effect sizes (e.g. Cohen's *d*, Pearson's *r*), indicating how they were calculated

Our web collection on statistics for biologists contains articles on many of the points above.

Software and code

Policy information about availability of computer code

Data collection	Fluorescence images were collected with Nikon Imaging Software (NISAdvanced, Nikon, Japan) with a CCD digital camera (CoolSNAPHQ2, Photometrics, Tucson, AZ, USA)Blood examination data was measured using a blood analyzer (COULTER Ac·T diff2, Beckman Coulter Inc., California, US), a flow cytometer (BD FACSCanto II) and a spectrophotometric microplate reader (bioTek Instruments, Inc.)
Data analysis	Origin 2021 was used for all statistical assessments.<br>ImageJ software version 1.45f was used for blots quantification and intensity calculation.<br>Flowjo_V10 was used for FCM images analysis.

For manuscripts utilizing custom algorithms or software that are central to the research but not yet described in published literature, software must be made available to editors and reviewers. We strongly encourage code deposition in a community repository (e.g. GitHub). See the Nature Portfolio guidelines for submitting code & software for further information.

Data

Policy information about availability of data

All manuscripts must include a data availability statement. This statement should provide the following information, where applicable:

- Accession codes, unique identifiers, or web links for publicly available datasets
- A description of any restrictions on data availability
- For clinical datasets or third party data, please ensure that the statement adheres to our policy

The data supporting the figures are provided with this paper as Source data file. The datasets are available from the corresponding author (T.J.H., email: tony.huang@duke.edu), and the data requests will be fulfilled within two weeks. Source data are provided with this paper.

Research involving human participants, their data, or biological material

Policy information about studies with human participants or human data. See also policy information about sex, gender (identity/presentation), and sexual orientation and race, ethnicity and racism.

Reporting on sex and gender	N/A
Reporting on race, ethnicity, or other socially relevant groupings	N/A
Population characteristics	N/A
Recruitment	N/A
Ethics oversight	N/A

Note that full information on the approval of the study protocol must also be provided in the manuscript.

Field-specific reporting

Please select the one below that is the best fit for your research. If you are not sure, read the appropriate sections before making your selection.

☒ Life sciences ☐ Behavioural & social sciences ☐ Ecological, evolutionary & environmental sciences

For a reference copy of the document with all sections, see nature.com/documents/nr-reporting-summary-flat.pdf

Life sciences study design

All studies must disclose on these points even when the disclosure is negative.

Sample size	Sample size was estimated to achieve about 90% power for detection of significant differences among groups based on means and standard deviations in preliminary studies. They were also consistent with the sample size of previously reported results.
Data exclusions	No data exclusion was performed.
Replication	All experiments were successfully performed with at least three technical replicates to ensure reproducibility.
Randomization	For blood experiments, samples were randomly allocated to corresponding experimental groups.
Blinding	Investigators were blinded to group allocation during experiments.

Reporting for specific materials, systems and methods

We require information from authors about some types of materials, experimental systems and methods used in many studies. Here, indicate whether each material, system or method listed is relevant to your study. If you are not sure if a list item applies to your research, read the appropriate section before selecting a response.

Materials & experimental systems

n/a	Involved in the study
☐	☒ Antibodies
☒	☐ Eukaryotic cell lines
☒	☐ Palaeontology and archaeology
☐	☒ Animals and other organisms
☒	☐ Clinical data
☒	☐ Dual use research of concern
☒	☐ Plants

Methods

n/a	Involved in the study
☒	☐ ChIP-seq
☐	☒ Flow cytometry
☒	☐ MRI-based neuroimaging

Antibodies

Antibodies used	PE anti-mouse/rat CD61 antibody (Biolegend, 104307)<br>APC anti-human CD3 antibody (Clone: SP34-2, BD Biosciences)<br>PE anti-MAMUA01 antibody (Clone: P12, Nonhuman Primate Reagent Resource, RRID: AB_2819290)
Validation	PE anti-mouse/rat CD61 antibody (Biolegend, 104307), APC anti-human CD3 antibody (Clone: SP34-2, BD Biosciences), PE anti-MAMUA01 antibody (Clone: P12, Nonhuman Primate Reagent Resource, RRID: AB_2819290): Flow cytometer (FCM), ee manufacturer's website for references.

Animals and other research organisms

Policy information about studies involving animals; ARRIVE guidelines recommended for reporting animal research, and Sex and Gender in Research

Laboratory animals	recipient animals (C57BL/6 mice) had been sensitized with BLAB/c donor skin transplantation
Wild animals	N/A
Reporting on sex	N/A
Field-collected samples	N/A
Ethics oversight	N/A

Note that full information on the approval of the study protocol must also be provided in the manuscript.

Plants

Seed stocks	N/A
Novel plant genotypes	N/A
Authentication	N/A

Flow Cytometry

Plots

Confirm that:

☒ The axis labels state the marker and fluorochrome used (e.g. CD4-FITC).

☒ The axis scales are clearly visible. Include numbers along axes only for bottom left plot of group (a 'group' is an analysis of identical markers).

☒ All plots are contour plots with outliers or pseudocolor plots.

☒ A numerical value for number of cells or percentage (with statistics) is provided.

Methodology

Sample preparation	To examine the levels of platelet in mouse blood after treated with ATAS, mouse blood were stained with PE anti-CD61 Antibody (Biolegend, 104307) for 2 min, and detected with Flow Cytometer (BD, Bioscience, USA).
Instrument	BD FACSCanto II
Software	Data analysis was done on FlowJo software (v10, FlowJo)
Cell population abundance	*Describe the abundance of the relevant cell populations within post-sort fractions, providing details on the purity of the samples and how it was determined.*
Gating strategy	Gating was performed based on identifying a distinct population in FSC vs SSC plots.

☐ Tick this box to confirm that a figure exemplifying the gating strategy is provided in the Supplementary Information.
